# Distribution and prognostic value of left ventricular global longitudinal strain in elderly patients with symptomatic severe aortic stenosis undergoing transcatheter aortic valve replacement

**DOI:** 10.1186/s12872-020-01791-9

**Published:** 2020-12-02

**Authors:** Jonas Agerlund Povlsen, Vibeke Guldbrand Rasmussen, Henrik Vase, Kaare Troels Jensen, Christian Juhl Terkelsen, Evald Høj Christiansen, Mariann Tang, Anders Lehmann Dahl Pedersen, Steen Hvitfeldt Poulsen

**Affiliations:** 1 Department of Cardiology, Aarhus University Hospital, Palle Juul-Jensens Boulevard 99, 8200 Aarhus N, Denmark; 2 Department of Cardiothoracic Surgery, Aarhus University Hospital, Palle Juul-Jensens Boulevard 99, 8200 Aarhus N, Denmark

**Keywords:** Global longitudinal strain, Aortic stenosis, TAVR, Echocardiography

## Abstract

**Aims:**

The aim of present study was to examine the preoperative prevalence and distribution of impaired left ventricular global longitudinal strain (LVGLS) in elderly patients with symptomatic aortic stenosis (AS) undergoing transcutaneous aortic valve replacement (TAVR) and to determine the predictive value of LVGLS on survival.

**Methods:**

We included 411 patients with symptomatic severe AS treated with TAVR during a 5-year period, where a baseline echocardiography including LVGLS assessment was available.

**Results:**

Mean age was 80.1 ± 7.1 years and aortic valve area (AVA) index 0.4 ± 0.1 cm^2^. 78 patients died during a median follow-up of 762 days. Mean left ventricular ejection fraction (LVEF) was 50 ± 13% and mean LVGLS was − 14.0%. LVEF was preserved in 60% of patients, while impaired LVGLS > − 18% was seen in 75% of the patients. Previous myocardial infarction, LVEF < 50%, LVGLS > − 14%, low gradient AS (< 4.0 m/s), tricuspid regurgitant gradient > 30 mmHg were identified as significant univariate predictors of all-cause mortality. On multivariate analysis LVGLS > − 14% (HR 1.79 [1.02–3.14], *p* = 0.04) was identified as the only independent variable associated with all-cause mortality. Reduced survival was observed with an impaired LVGLS > − 14% in the total population (*p* < 0.002) but also in patients with high AS gradient with preserved LVEF. LVGLS provided incremental prognostic value with respect to clinical characteristics, AVA and LVEF (χ^2^ 19.9, *p* = 0.006).

**Conclusions:**

In patients with symptomatic AS undergoing TAVR, impaired LVGLS was highly prevalent despite preserved LVEF. LVGLS > − 14% was an independent predictor of all-cause mortality, and survival was reduced if LVGLS > − 14%.

## Background

Surgical aortic valve replacement (SAVR) is considered gold standard therapy in severe aortic stenosis (AS) alleviating symptoms, improving quality of life and prolonging survival [1]. Transcatheter aortic valve replacement (TAVR) is often the preferred strategy for treatment of symptomatic severe AS in elderly patients with intermediate and high perioperative risk [2, 3]. The clinical characteristics of patients undergoing TAVR differ significantly from those undergoing SAVR in terms of age, frailty, comorbidity and coexisting cardiac disease such as transthyretin amyloidosis.

The current guidelines recommend SAVR or TAVR for severe AS once symptoms occur or when left ventricular ejection fraction (LVEF) is < 50% [4, 5]. To determine whether patients are truly asymptomatic or even symptomatic can be challenging in the elderly and often frail AS patients considered for TAVR. Exercise testing, although recommended in patients with unclear symptom status, is often not a diagnostic option as many patients are unable to perform this test [6].

The assessment of left ventricular (LV) systolic function by EF is considered a central parameter for timing of intervention. However, LVEF is often preserved until late in the disease even after symptoms, progression of AS severity and LV hypertrophy have developed indicating lack of accuracy in detecting subtle changes of myocardial performance [7]. In addition, LVEF has in recent reports failed to predict outcome in asymptomatic and symptomatic AS patients with a low-intermediate risk profile [8, 9]. In contrast, LV global longitudinal systolic strain (LVGLS) assessment has been demonstrated to detect subtle changes of LV systolic function, good correlation to symptoms and independent prognostic value in asymptomatic AS [7–11]. LVGLS has also proven a more reliable and reproducible parameter than standard 2-dimensional echocardiographic derived LVEF [12]. Although LVGLS as well as LVEF is pre-and afterload dependent, the sensitivity of LVGLS is sufficient to unmask subclinical myocardial dysfunction. In patients with preserved LVEF impaired LVGLS is a powerful predictor of outcome in the general AS population [8, 11]. However, little is known about the prognostic value of LVGLS among the elderly symptomatic AS patients undergoing TAVR—a group of patients that currently accounts for up to two-thirds of all AS patients receiving valve intervention therapy.

The aims of present study was to determine the preoperative LVGLS status among symptomatic elderly AS patients undergoing TAVR with an intermediate to high risk profile with respect to survival, and secondly to determine the predictive value of LVGLS in the overall AS population but also in patients with high gradient AS with preserved LVEF recognized as the most prevalent AS subtype.

## Methods

### Study population

This study is an observational cohort study of symptomatic AS patients, who underwent TAVR at our institution. 681 procedures were performed from July 1st 2012 to June 30th 2017. 411 patients were included. We excluded patients who underwent a valve in valve intervention, had intervention done on non-aortic valves and had unavailable or inadequate preoperative echocardiographies. A consort diagram can be seen in Additional file 1: Figure S1.

Data were collected from the electronic patient journal (MidtEPJ, Systematic, Aarhus, DK) and The Western Denmark Heart Registry.

### Echocardiography

Analysis was performed on the preoperative transthoracic echocardiography (TTE). Image data was examined using Echopac version 202 (GE Healthcare, Milwaukee, Wisconsin, USA) according to current guidelines [13].

We measured peak systolic 2D LVGLS using Automated Function Imaging (AFI) with a frame rate > 55 frames-per-second from the standard apical views. 3D echocardiography was used in patients with atrial fibrillation to acquire simultaneous triplane apical views. A 17 segments model was automatically generated and weighted averages of overall peak systolic strain, basal-, mid- and apical segments were calculated.

LVEF was measured with Simpson's biplane method. Left atrial (LA) volume was assessed with biplane area-length method in apical two and four chamber views. LA volume was corrected for body surface area (BSA) to calculate left atrial volume index (LAVI). Left ventricular outflow tract (LVOT) dimension was measured in the parasternal long axis view. Peak – and mean velocity of LVOT and LVOT velocity time integral (VTI) were measured with pulsed-wave Doppler in apical five-chamber view. Stroke volume index (SVI) was calculated by multiplying LVOT VTI with calculated LVOT area indexed by BSA. Peak and mean gradients over the aortic valve were measured using continuous-wave Doppler. Velocities were converted to gradients by using the modified Bernoulli Formula. The aortic valve area (AVA) was calculated by the continuity equation and indexed (AVAI) by BSA. The echocardiographic analysis was performed by a single reader (JAP) who was blinded to the clinical- and survival status.

### AS subgroup definitions

Patients were analysed in guidelines specified subgroups, where high maximal gradient (HG) was defined as ≥ 4 m/s and normal EF (NEF) as ≥ 50%. Low gradient (LG) was defined as < 4 m/s. LVGLS were dichotomized with predefined cut-off of − 14% based on previous studies [8].

### Statistics

Continuous data are presented as mean ± standard deviation (SD) or median ± interquartile range (IQR) depending on normal or non-normal distribution. Categorical data are presented as percentages. Normality of data distribution was assessed using Q–Q plots and histograms. Statistical differences between groups were assessed using student's *t*-test for normal distributed data, Mann–Whitney *U* test for skewed distributed data and chi-square test for categorical variables.

Survival analysis was calculated by Kaplan–Meier estimates. Differences between groups were compared using log-rank test and hazard ratios were determined with Cox proportional hazard regression models.

Variables considered as potential predictors for multivariate modeling were selected for uni- and multivariate analyses. The incremental value of LVGLS was assessed in three modeling steps. The first step consisted of fitting a multivariate model of clinical parameters. LVEF and aortic valve area were then added. Finally, LVGLS was included. The change in overall log likelihood ratio χ^2^ was used to assess the increment of predictive power at each step.

*P* values < 0.05 were considered statistically significant. Data were analyzed using STATA16 (StataCorp LP, Texas, College Station, USA).

## Results

### Baseline clinical parameters

The study consisted of 411 AS patients undergoing TAVR (54% men) with a median age of 80 years. Table 1 summarizes the clinical characteristics of the patients including the type of aortic valve prosthesis implanted. We compared data for patients categorized as survivors (81%) to non-survivors (19%) after a median follow-up of 762 days (IQR 590 days).

**Table 1:** Clinical characteristics of all patients and in survivors or non-survivors

	All (n = 411)	Survivor (n = 333)	Non-survivor (n = 78)	*P* value Survivor versus non-survivor
Age (years)	80.1 ± 7.1	79.9 ± 7.2	81.0 ± 6.2	0.24
Female sex	46.0% (189/411)	46.3 (154/333)	44.9 (35/78)	0.83
BMI (kg/m^2^)	26.5 ± 4.5	26.7 ± 4.6	25.7 ± 4.0	0.09
Body surface area (m^2^)	1.80 ± 0.20	1.85 ± 0.20	1.84 ± 0.21	0.63
Systolic blood pressure (mmHg)	141 ± 23	142 ± 22	142 ± 29	0.91
Diastolic blood pressure (mmHg)	75 ± 12	74 ± 12	76 ± 13	0.64
Creatinine clearance (ml/min)	60 (IQR 28)	62 (QR 28)	57 (IQR 27)	0.25
Eurolog II (%)	3.1 (IQR 3.4)	2.9 (IQR 2.8)	4.3 (IQR 5.7)	0.0001
Hemodialysis	1.0% (4/411)	0.9% (3/333)	1.3% (1/78)	0.76
Previous myocardial infarction	12.0% (46/385)	10.4% (32/309)	18.4% (14/76)	0.05
Hypertension	72.5% (271/374)	73.8% (222/301)	67.1% (49/73)	0.26
COPD	15.8% (65/411)	15.0% (50/333)	19.2% (15/78)	0.36
PAD	14.4% (59/410)	13.3% (44/332)	19.2% (15/78)	0.18
DM2	18.3% (68/372)	18.3% (55/301)	18.3% (13/71)	0.99
NYHA I–II	22.5% (92/409)	24.5% (81/331)	14.1% (11/78)	0.0001
NYHA III	68.7% (281/409)	69.5% (230/331)	65.4% (51/78)	
NYHA IV	8.8% (36/409)	6.0% (20/331)	20.5% (16/78)	
*Medications*				
Statins	59.0% (222/379)	59.7% (181/303)	56.2% (41/73)	0.58
Beta blockers	50.1% (204/407)	48.6% (161/331)	56.6% (43/76)	0.21
Calcium antagonists	25.0% (102/408)	26.2% (87/332)	19.7% (15/76)	0.24
ACE inhibitors/ARB	33.3% (136/408)	34.0% (113/332)	30.3% (23/76)	0.53
Anticoagulant treatment	26.2% (107/408)	24.4% (81/332)	34.2% (27/76)	0.08
Thrombocyte inhibitors	40.7% (166/408)	42.8% (142/332)	31.6% (24/76)	0.07
*Valve characteristics*				
Valve type				
Edwards S3	80.3% (330/411)	79.9% (266/333)	82.1% (64/78)	0.73
Edwards XT	9.7% (40/411)	9.3% (31/333)	11.5% (9/78)	
Evolute	1.9% (8/411)	2.4% (8/333)	0% (0/78)	
Lotus	1.7% (7/411)	1.8% (6/333)	1.3% (1/78)	
Others	6.4% (26/411)	6.6% (22/333)	5.1% (4/78)	
Valve size (mm)	26.1 ± 2.4	26.0 ± 2.4	26.5 ± 2.3	0.10

BMI, Body Mass Index; COPD, Chronic Obstructive Pulmonary Disease; PAD, Peripheral Artery Disease; DM2, Type 2 Diabetes Mellitus; NYHA, New York Heart Association Class; ACE, Angiotensin Converting Enzyme; ARB, Angiotensin Receptor Blocker

Presented as mean ± SD, median (interquartile range) or fraction (%)

### LV systolic function and AS valve characteristics

**Fig. 1:**
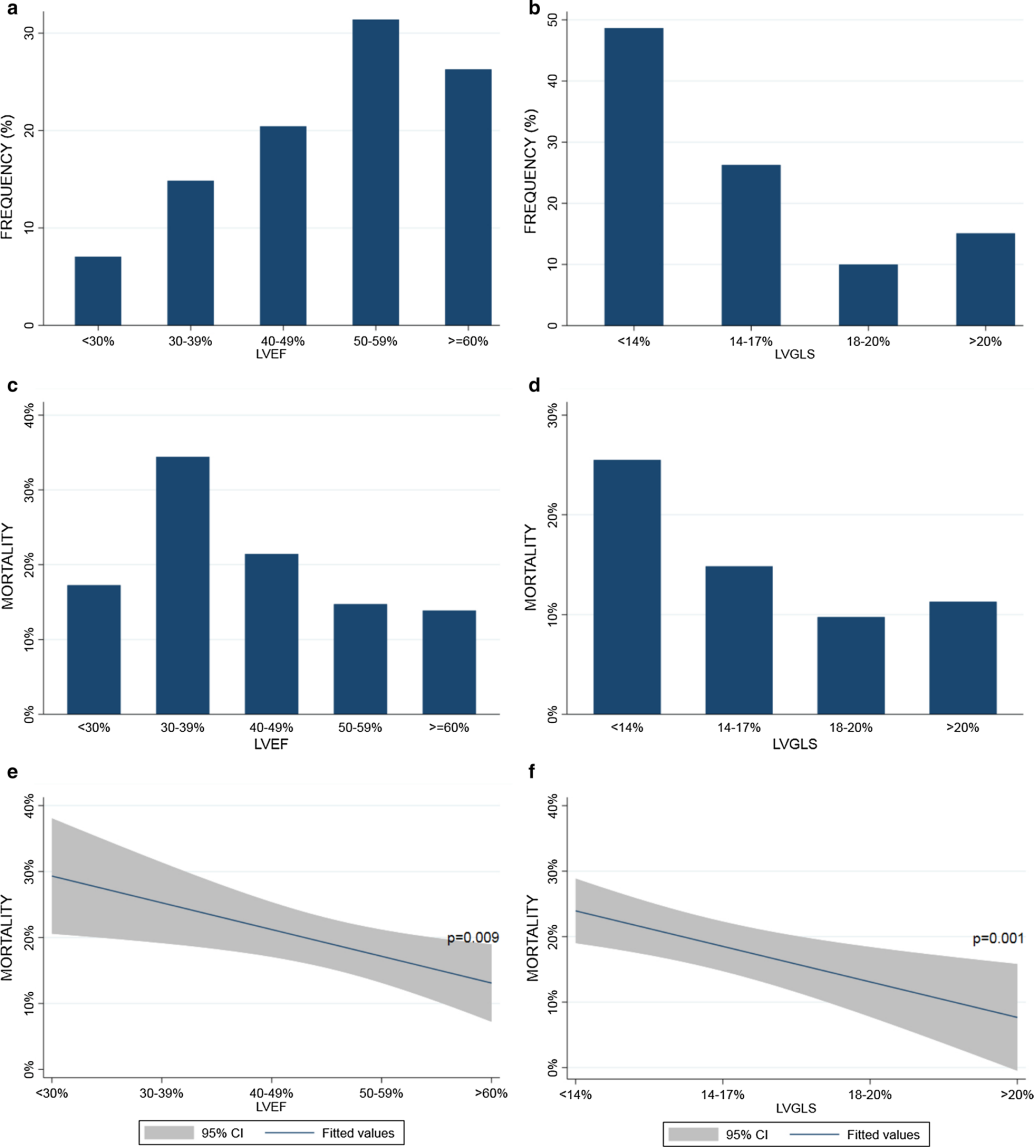
LVEF and LVGLS distribution and their correlation with overall mortality. Distribution of LVEF (**a**) and LVGLS (**b**) as a function of predefined intervals demonstrated that the majority of patients had preserved LVEF but impaired LVGLS. There was a significant correlation between mortality and LVEF (**c** + **e**) as well as LVGLS (**d** + **f**), and the latter reached highest statistical significance

Echocardiographic data are presented in Table 2 for all patients, survivors and non-survivors. Median LVEF was 52% (IQR 19%) and LVGLS − 14.1% (IQR 7.3%). The distribution of LVEF and LVGLS according to predefined intervals in the overall AS population are shown in Fig. 1a, b. LV systolic parameters such as LVEF, LVGLS, and SVI were significantly reduced in the non-survivor group as compared to the survivors. The AS severity, determined by calculated AVA, AVAI and mean gradient, were comparable between non-survivors and survivors. Peak aortic valve velocity was significantly higher among survivors.

**Table 2:** Echocardiographic characteristics of all patients and in survivors or non-survivors

	All (n = 411)	Survivor (n = 333)	Non-survivor (n = 78)	*P* value Survivor versus non-survivor
*Left atrium and ventricle*				
EF (%)	50 ± 13	51 ± 12	47 ± 13	0.007
LVGLS (%)	− 14.0 ± 5.2	− 14.6 ± 4.9	− 12.4 ± 5.2	0.0007
LVGLS Basal (%)	− 10.1 ± 4.1	− 10.3 ± 4.1	− 9.0 ± 4.2	0.009
LVGLS Mid (%)	− 13.7 ± 4.9	− 14.0 ± 4.9	− 12.1 ± 4.9	0.002
LVGLS Apex (%)	− 19.8 ± 8.5	− 20.5 ± 8.3	− 17.0 ± 9.0	0.001
LVOT diameter (cm)	2.0 ± 0.2	2.0 ± 0.2	2.0 ± 0.2	0.36
LVOT Vmax (m/s)	0.9 ± 0.2	0.9 ± 0.2	0.9 ± 0.2	0.12
LVOT VTI (cm)	21.8 ± 5.7	22.2 ± 5.8	19.7 ± 5.2	0.0004
Stroke Volume Index (mL/m^2^)	36.5 ± 10.1	37.1 ± 10.3	33.8 ± 9.1	0.009
Left atrial volume index (mL/m^2^)	48 ± 18	46 ± 16	55 ± 23	0.0002
*Aortic valve*				
AV Vmax (m/s)	4.1 ± 0.8	4.1 ± 0.7	3.9 ± 0.9	0.02
AV Peak Gradient (mmHg)	70 ± 28	71 ± 25	64 ± 28	0.05
AV Mean Gradient (mmHg)	39 ± 16	40 ± 16	36 ± 18	0.07
AV VTI (cm)	97.7 ± 23.3	99.3 ± 22.9	90.7 ± 23.7	0.003
AV Area (cm^2^)	0.7 ± 0.3	0.7 ± 0.2	0.7 ± 0.2	0.90
AV Area Index (cm^2^/m^2^)	0.4 ± 0.1	0.4 ± 0.1	0.4 ± 0.1	0.78
*Right ventricle*				
TR gradient > 30 mmHg	40.3%	37.8%	51.4%	0.03

EF, Ejection Fraction; LVGLS, Left Ventricular Global Longitudinal Strain; LVOT, Left Ventricular Outflow Tract; AV, Aortic Valve; Vmax, Maximal Velocity; VTI, Velocity Time Integral; TR, Tricuspid Regurgitation

Presented as mean ± SD or fraction (%)

### High gradient AS with preserved LVEF subgroup analysis

In subgroup analysis we identified 155 patients with HG AS and preserved LVEF. These patients were divided into two groups according to LVGLS ≤ − 14% or > − 14%. Clinical characteristics were comparable between groups.

In Table 3 the clinical characteristics and echocardiographic parameters of the two subgroups are presented. In patients with LVGLS ≤ − 14% both LVEF and LVGLS were in the normal range, whereas patients with LVGLS > − 14% had a significantly lower LVEF but within normal range. No differences were observed in aortic valve gradients or clinical characteristics. In the HG NEF LVGLS subgroup no clinical or echocardiographic variables correlated with overall mortality in uni- and multivariate analysis.

**Table 3:** Echocardiographic characteristics in high-gradient AS with preserved LVEF according to LVGLS

	HG NEF LVGLS ≤ − 14% (n = 118)	HG NEF LVGLS > − 14% (n = 37)	*P* value
*Clincal characteristics*			
Age (years)	80.5 ± 7.5	80.1 ± 6.7	0.80
Female sex	57.6% (68/118)	56.8% (21/37)	0.93
Creatinine clearance (ml/min)	58 (IQR 35)	62 (IQR 32)	0.61
Eurolog II (%)	2.4 ± 2.1	2.2 ± 2.4	0.98
Previous myocardial infarction	3.6% (4/112)	9.4% (3/32)	0.37
Hypertension	68.9% (73/106)	74.2% (23/31)	0.74
DM2	15.5% (16/103)	21.2% (7/32)	0.56
NYHA I–II	26.3% (31/118)	22.2% (8/36)	0.86
NYHA III	70.3% (83/118)	75.0% (27/36)	
NYHA IV	3.4% (4/118)	2.8% (1/36)	
*Left atrium and ventricle*			
EF (%)	60 ± 6	57 ± 4	0.004
LVGLS (%)	− 18.4 ± 2.8	− 12.4 ± 1.3	< 0.0001
LVOT Diameter (cm)	2.0 ± 0.1	1.9 ± 0.2	0.16
LVOT Vmax (m/s)	1.0 ± 0.2	1.0 ± 0.2	0.11
LVOT VTI (cm)	25.3 ± 4.9	23.4 ± 6.0	0.06
Stroke Volume Index (mL/m^2^)	42.7 ± 9.8	37.5 ± 9.5	0.005
Left Atrial Volume Index (mL/m^2^)	44 ± 15	51 ± 16	0.04
*Aortic valve*			
AV Vmax (m/s)	4.7 ± 0.5	4.7 ± 0.6	0.90
AV Peak Gradient (mmHg)	89 ± 19	89 ± 20	0.85
AV Mean Gradient (mmHg)	50 ± 14	50 ± 15	0.92
AV Area (cm^2^)	0.7 ± 0.3	0.6 ± 0.2	0.03
AV Area Index (cm^2^/m^2^)	0.4 ± 0.1	0.3 ± 0.1	0.01

HG, High Gradient; NEF, Normal Ejection Fraction; EF, Ejection Fraction; LVGLS, Left Ventricular Global Longitudinal Strain; LVOT, Left Ventricular Outflow Tract; AV, Aortic Valve; Vmax, Maximal Velocity

Presented as mean ± SD

### Determinants of all-cause mortality in AS after TAVR

Figure 1c–f demonstrate the relationship between the degree of LVEF—and LVGLS impairment with overall mortality.

**Fig. 2:**
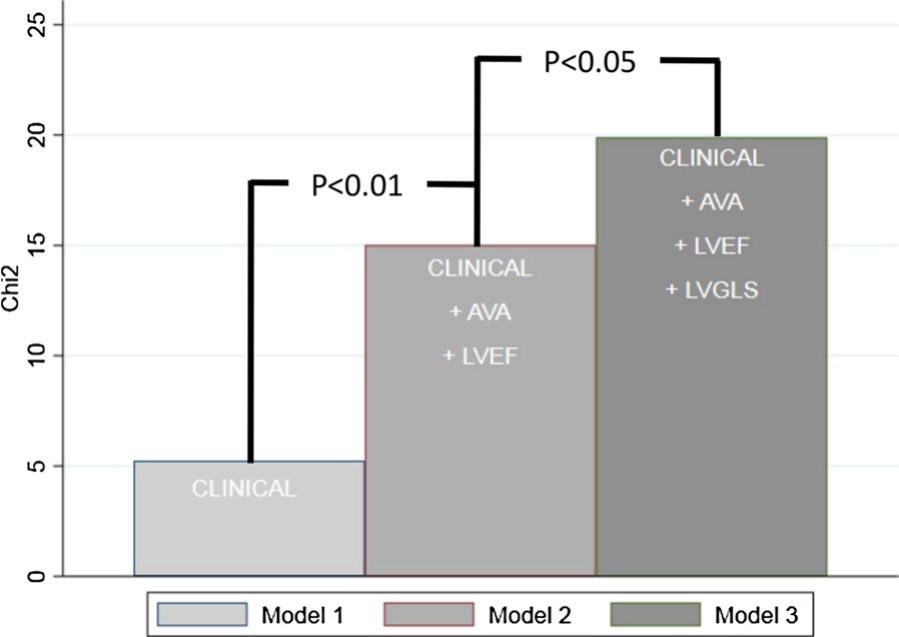
Mortality prediction models. There was an incremental value of assessment of LVGLS in predicting mortality. Addition of aortic valve area (AVA) and left ventricular ejection fraction (LVEF) significantly improved model 1 which included clinical variables (age, sex, previous myocardial infarction and creatinine clearance). Further improvement was achieved by addition of LVGLS to model 2

Correlation between clinical and echocardiographic variables of LV systolic function, AS severity and all-cause mortality after TAVR was examined by univariate analysis as shown in Table 4. Previous myocardial infarction, LVEF < 50%, LVGLS > − 14%, LG and tricuspid regurgitant gradient > 30 mmHg were identified as significant correlates to all-cause mortality. LVGLS worsening by [1%] was also identified as a significant correlate (HR 1.07 [1.03; 1.12], *p* = 0.002). In multivariate analysis LVGLS > − 14% (HR 1.79 [1.02–3.14], *p* = 0.04) was the only independent variable associated to all-cause mortality and provided incremental prognostic value to AVA, LVEF and selected clinical characteristic (χ2 = 19.9, *p* = 0.006) (Fig. 2). The multivariate analysis was corrected for variables associated with left ventricular function that reached statistical significance in the univariate analyses (LVGLS, LVEF and LG). When correcting for clinical variables (age, sex, creatinine clearance, NYHA class and previous myocardial infarction) no variables reached statistical significance.

**Table 4:** Univariate and multivariate analysis for all-cause mortality after TAVR

	Hazard ratio	*P* value
*Univariate analysis*		
Age	1.02 [0.98; 1.05]	0.32
Female sex	0.87 [0.56; 1.37]	0.87
Creatinine clearance < 60 ml/min	1.36 [0.85; 2.17]	0.20
Previous MI	1.79 [1.00; 3.21]	0.05
EF < 50%	1.69 [1.09; 2.66]	0.02
LVGLS > − 14%	2.05 [1.28; 3.26]	0.003
Aortic valve area	1.11 [0.37; 3.37]	0.85
Aortic valve mean gradient	0.98 [0.97;1.00]	0.06
AS low gradient (< 4 m/s)	1.75 [1.11; 2.75]	0.02
TR gradient > 30 mmHg	1.49 [1.07; 2.08]	0.02
*Multivariate analysis*		
LVGLS > − 14%	1.79 [1.02; 3.14]	0.04
EF < 50%	1.12 [0.65; 1.92]	0.68
AS low gradient (< 4 m/s)	1.56 [0.98; 2.48]	0.04

MI, Myocardial Infarction; EF, Ejection Fraction; LVGLS, Left Ventricular Global Longitudinal Strain; AS, Aortic Stenosis; TR, Tricuspid Regurgitation, Presented as Hazard ratios [95% CI]

### Sensitivity analysis

We omitted all patients with NYHA class IV in a sensitivity analysis to evaluate their contribution to the main results in the survivor and non-survivor groups. The results are shown in Additional file 2: Table S1. Overall, measures of left ventricular function improved and comorbidity burden, estimated by Eurolog II, score decreased. Differences between groups decreased but remained significant except for LVEF and AV peak velocity.

### Survival outcome and LVGLS

**Fig. 3:**
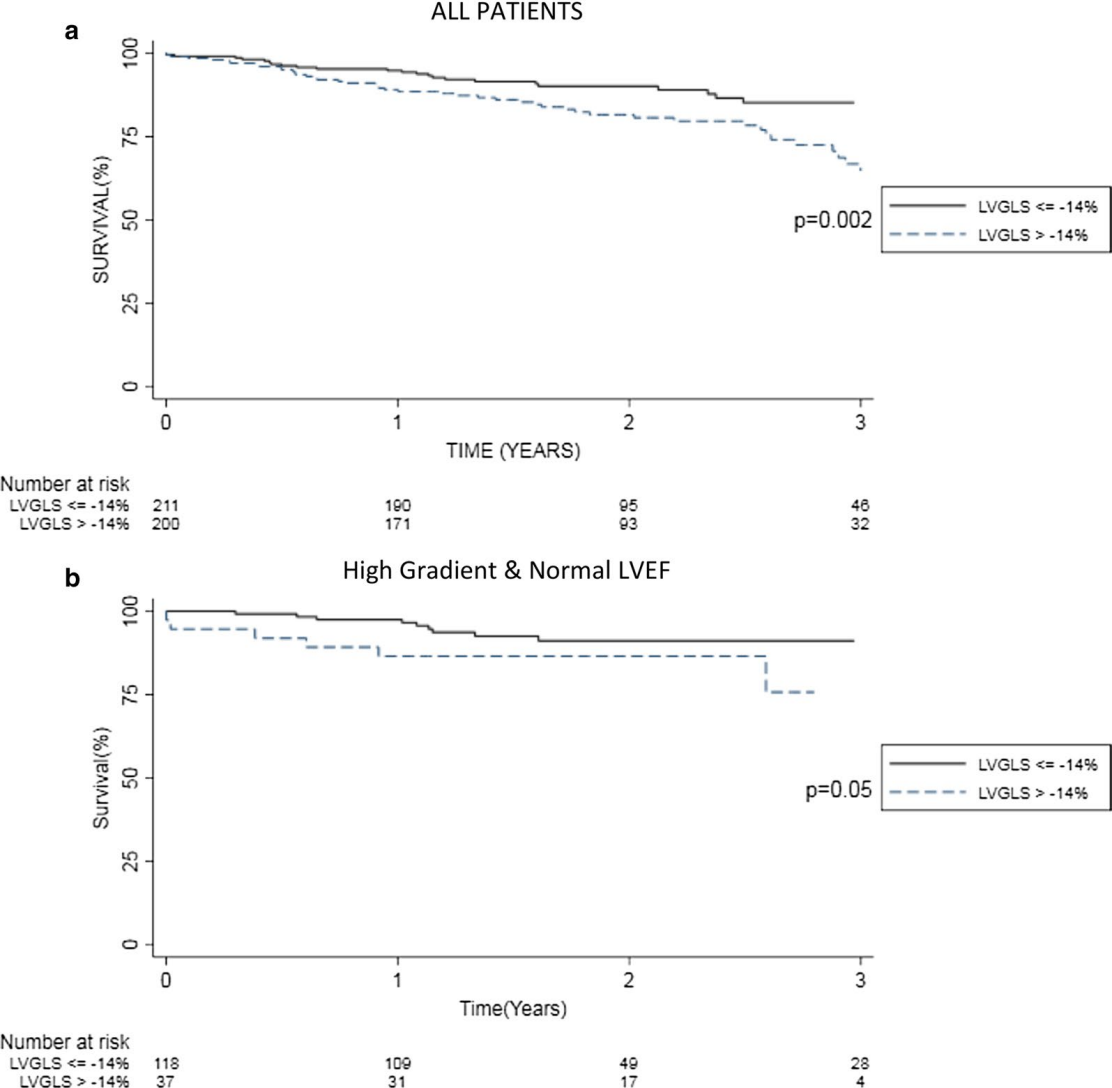
Survival by LVGLS overall and in patients with HG and NEF AS. Kaplan–Meier estimates of cumulative survival in all patients (**a**) and in a subgroup of patients with high gradient (> 4 m/s) aortic stenosis and left ventricular ejection fraction ≥ 50% (**b**) with LVGLS > and ≤ − 14%, respectively. Patients with LVGLS ≤ − 14% had superior survival compared with patients impaired LVGLS > − 14%

The overall mortality after 12, 24 and 36 months was 8.2%, 14.3% and 23.5. Patients with LVGLS > − 14% had higher all-cause mortality as compared to patients with LVGLS ≤ − 14% (Fig. 3a).

Patients with HG NEF AS with LVGLS > − 14% had a higher mortality compared to patients with a LVGLS ≤ − 14% (Fig. 3b).

The clinical characteristics of patients with impaired and preserved LVGLS are shown in Additional file 2: Table S2. Patients with LVGLS > − 14% were more symptomatic in terms of NYHA class, more comorbid (Eurolog II score, COPD and previous myocardial infarction) and received more medication (ACE-inhibitors, calcium antagonists and beta-blockers).

## Discussion

The main findings of this study investigating elderly symptomatic patients with severe AS undergoing TAVR are: (1) LVEF was preserved in 60% of the patients despite advanced symptoms in contrast to LVGLS that was abnormal in 75% (> − 18%) and at least moderately decreased (> − 14%) in approximately half of the patients; (2) a highly significant correlation between LVGLS and all-cause mortality was identified; (3) LVGLS > − 14% was identified as an independent predictor of all-cause mortality and patients with a LVGLS > − 14% had reduced long-term survival as compared to patients with LVGLS ≤ − 14%; (4) patients with high gradient AS with preserved LVEF but LVGLS > − 14% had reduced survival as compared to patients with LVGLS ≤ − 14%.

The main factors that determine the timing for SAVR or TAVR intervention according the current AHA/ESC guidelines are symptoms and/or LV systolic dysfunction in terms of LVEF < 50% [4, 5]. In recent years TAVR treatment of severe AS is routinely offered in many centres to elderly patients with intermediate to high—and in some cases also patients at low preoperative risk. The population of patients above 75 years with severe AS treated with TAVR is increasing and, according to the Danish Heart Registry, accounts for two-thirds of all aortic valve interventions in 2018. The interpretation of symptoms in the elderly with co-morbidities and decreased physical activity level can be challenging and may result in failure to recognise symptoms or late reporting of symptoms [14]. Evaluation of LV systolic function is therefore of particular interest and importance in order to refer the patient for timely valve intervention before potential irreversible LV dysfunction occurs. In both asymptomatic and symptomatic severe AS the progressive AVA reduction leads to increasing afterload, which is usually accompanied by compensatory LV hypertrophy. This LV remodelling process tends to normalize the LV wall stress and maintenance of LVEF. A normal LVEF is often present until late in the disease stage. At this stage a mismatch develops between afterload and inadequate LV hypertrophy response, which is independent of symptom status as seen in the present study where 60% of the all patients had preserved LVEF but severe symptoms (78% were in NYHA class III or IV). In contrast to LVEF, myocardial strain analysis including assessment of LVGLS has been demonstrated to be able to detect subclinical myocardial dysfunction in a wide range of AS severities with LVEF 60% [11]. The LV systolic dysfunction determined by LVGLS seems to appear first in the subendocardial layer and progresses transmurally with increasing severity of AS independent of LVEF [11]. In addition, patients with symptoms seem to have more impaired levels of LVGLS as compared to asymptomatic patients. Recently, a meta-analysis of LVGLS in 1067 asymptomatic AS patients with LVEF 50% (median LVEF 63.5%) and AVAI of 0.49 cm^2^ demonstrated an average LVGLS of − 16.2% [15]. From other studies LVGLS in severe AS with preserved LVEF has been reported to be − 15% on average and with lower absolute values if symptoms were present and/or LVEF was < 50% [8, 11, 16]. In our study we noted a lower mean LVGLS of − 14.0%, which might be explained by a higher degree of AS severity determined by AVA/AVAI, a lower mean LVEF of 50% and presence of more advanced symptoms as compared to the aforementioned studies. The majority of AS patients in our study had an abnormal LVGLS > − 18% in contrast to normal LVGLS ≤ − 20% which was found in only 15% of the patients. Preserved LVGLS is most often seen in patients with aortic valve sclerosis or mild AS and is noted in less than 15% of patients with severe asymptomatic AS with preserved LVEF [11, 15].

Overall, assessment of LVGLS seems to be a suitable tool for monitoring LV systolic function in AS enabling detection of early myocardial contractile dysfunction. Furthermore, LVGLS relates to both AS severity and progression of the AS severity in contrast to LVEF. LVGLS is easily calculated, has a good feasibility and has an inter-and intra-variability of 8% and 5% that is even better than bi-plane LVEF analysis of 10% and 8%, respectively [12].

Abnormal and worsening LVGLS in AS is likely to reflect several factors such as inadequate compensatory LV hypertrophy, subendocardial ischemia, neurohumoral up-regulation, myocyte degeneration and replacement fibrosis [17–19]. Increased interstitial myocardial fibrosis has been reported in AS with preserved LVEF but with impaired longitudinal systolic function [19]. Presence of increased myocardial fibrosis detected by staining of LV biopsies taken during SAVR operation was associated with significantly higher serum Nt-pro-brain natriuretic peptide (NT-pro-BNP), higher LV mass index and impaired LVGLS [20]. Late gadolinium enhancement (LGE) by cardiac magnetic resonance (CMR) imaging detects myocardial fibrosis and a positive LGE-CMR has been shown to be present in 49% of patients with AS. Myocardial fibrosis identified by LV biopsy staining or LGE-CMR independently predicts mortality [20–22]. Recently, co-existing transthyretin wild type cardiac amyloidosis (ATTRwt) has been demonstrated in AS patients undergoing TAVR with a prevalence of 16% [23]. Myocardial amyloid deposition impairs LV longitudinal systolic function and an apical-basal strain pattern is often present. The presence of ATTRwt in AS reduces LVGLS and is likely to affect prognosis after AVR.

Marwick et al. have previously demonstrated that LVGLS is an independent predictor of death or AV replacement in asymptomatic severe AS patients with LVEF 50%. Baseline LVGLS > − 15% was associated with significantly increased mortality [24]. In a recent meta-analysis by Magne et al. it was demonstrated that impaired LVGLS (> − 14.7%) was associated with reduced survival in patients with significant asymptomatic AS with LVEF 50% and even in patients with LVEF 60% [15]. Impaired LVGLS (> − 18.2%) has been shown to predict disease progression with development of symptoms and need for AV replacement in a population with asymptomatic severe AS with preserved LVEF [7]. In two studies including asymptomatic as well as symptomatic patients with a wide range of AS severities and preserved LVEF, LVGLS was identified as a strong independent predictor of all-cause mortality. Patients with LVGLS > − 14% had a reduced survival [8, 25]. Furthermore, in a recent study Al-Rashid et al. demonstrated that baseline LVGLS correlate significantly with postprocedural outcomes in 150 consecutive patients undergoing TAVR [26]. In addition, LVEF did not show any predictive value in terms of all-cause mortality, AV replacement or development of cardiac symptoms in the studies referred to above. The lack of predictive value of LVEF of all-cause mortality in asymptomatic AS patients has previously been documented in a larger series of AS patients with prolonged follow-up [27].

LVGLS > − 14% was an independent predictor of all-cause mortality and a strong association between severity of LVGLS impairment and mortality was noted. A risk model demonstrated additive prognostic value of LVGLS to clinical characteristics, AVA and LVEF. LVGLS seems a more reliable parameter than LVEF for evaluating myocardial function and prognosis in both asymptomatic and symptomatic AS patients with a wide range of severities and ages. Assessment of LVGLS in AS patients could potentially contribute to a more optimal decision process against a SAVR or TAVR, and the current published data on the subject should be considered implemented in future guidelines.

### Limitations

The present study is limited by its retrospective design and because it was performed as a single centre study in a tertiary cardiovascular referral centre. The latter might induce selection bias as the included patients only account for patient referred for AV replacement evaluation at our institution. Furthermore, patients selected for conservative treatment are not included in the analysis.

LVGLS may be influenced by a variety of pathologies including myocardial ischemia, previous myocardial infarction, diabetes mellitus, hypertension and amyloidosis. We do not report data on the specific extent of coronary artery disease. No systematic screening or investigations for ATTRwt was performed. Although, LVGLS does not detect the specific cause of the myocardial dysfunction, the parameter is not limited to being an independent marker of adverse risk in AS.

We only report biochemical measures of kidney function in this study, as no other laboratory data were available in our database. Measures of myocardial injury (e.g. troponins) and heart failure (NT-proBNP) could potentially provide predictive capabilities of our endpoint and affect other parameters. Short-term complications such as moderate-to-severe paravalvular leak [28], new-onset left-bundle-branch-block [29] and persistent severe mitral regurgitation [30] are also potential contributors to a poor long-term outcome. We do not have any data on their role in the present study.

## Conclusions

LVGLS detected myocardial systolic dysfunction in the majority of elderly patients with advanced symptomatic AS undergoing TAVR, even though preserved LVEF was noted in 60% of the patients. The level of LVGLS impairment was significantly associated to increased mortality and LVGLS > − 14% was an independent predictor of all-cause mortality. Survival was significantly reduced if baseline LVGLS was > − 14% in the total population as well as among patients with high gradient AS with preserved LVEF.

The present and previous published data emphasize the importance of assessment of LVGLS in the evaluation of AS patients independent of patient age, symptoms and LVEF. These findings support the consideration of implementing LVGLS in future valvular heart disease recommendations.

## Supplementary information

**Additional file 1: Figure S1**. Consort diagram. 681 TAVR procedures were performed between July 2012 and June 2017. A total of 411 patients were included in the analysis.

**Additional file 2: Table S1**. Sensitivity analysis excluding NYHA IV patients. **Table S2**. Clinical characteristics of patients with preserved and impaired LVGLS.

## Data Availability

The datasets generated and/or analysed during the current study are not publicly available due to their patient referable character, thereby compromising individual privacy, but are available from the corresponding author on reasonable request.

## Abbreviations

2D: Two-dimensional
3D: Three-dimensional
AS: Aortic stenosis
AFI: Automated Function Imaging
AVA: Aortic valve area
AVAI: Aortic valve area index
ATTRwt: Transthyretin wild type cardiac amyloidosis
BSA: Body surface area
CI: Confidence interval
CMR: Cardiac magnetic resonance
HG: High gradient
HR: Heart rate
IQR: Interquartile range
LA: Left atrium
LAVI: Left atrial volume index
LV: Left ventricle
LVEF: Left ventricle ejection fraction
LG: Low gradient
LGE: Late gadolinium enhancement
LVGLS: Left ventricle global longitudinal strain
LVOT: Left ventricular outflow tract
NEF: Normal ejection fraction
NYHA: New York Heart Association
NT-pro-BNP: Nt-pro-brain natriuretic peptide
SAVR: Surgical aortic valve replacement
SD: Standard deviation
SVI: Stroke volume index
TAVR: Transcatheter aortic valve replacement
TTE: Transthoracic echocardiography
VTI: Velocity time integral
