# Supplementary figures and images for: Distribution and prognostic value of left ventricular global longitudinal strain in elderly patients with symptomatic severe aortic stenosis undergoing transcatheter aortic valve replacement
Source: BMC Cardiovasc Disord. 2020 Dec 2;20:506. doi: 10.1186/s12872-020-01791-9 (PMC7709407; doi:10.1186/s12872-020-01791-9)

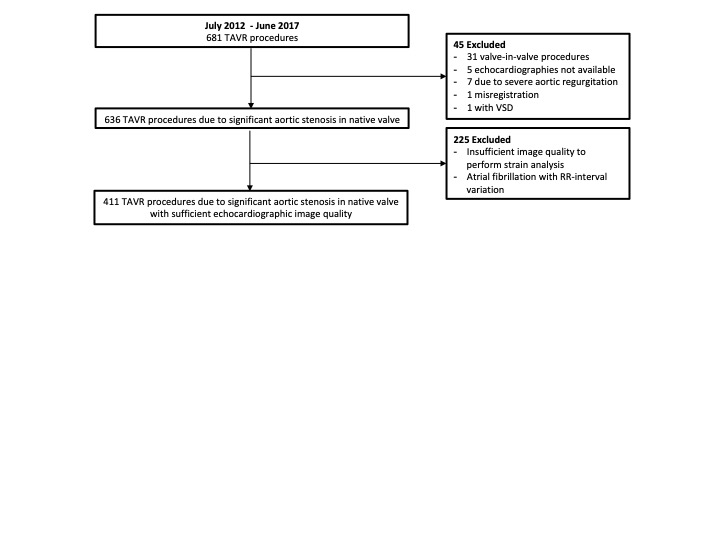

Supplement: Supplementary file 1 — Additional file 1: Figure S1. Consort diagram. 681 TAVR procedures were performed between July 2012 and June 2017. A total of 411 patients were included in the analysis. [file 12872_2020_1791_MOESM1_ESM.jpg]
