# Supplementary material for: Distribution and prognostic value of left ventricular global longitudinal strain in elderly patients with symptomatic severe aortic stenosis undergoing transcatheter aortic valve replacement
Source: BMC Cardiovasc Disord. 2020 Dec 2;20:506. doi: 10.1186/s12872-020-01791-9 (PMC7709407; doi:10.1186/s12872-020-01791-9)
Supplement: Supplementary file 2 — Additional file 2: Table S1. Sensitivity analysis excluding NYHA IV patients. Table S2. Clinical characteristics of patients with preserved and impaired LVGLS. [file 12872_2020_1791_MOESM2_ESM.docx]

**Additional file 2: Table S1. Sensitivity analysis excluding all NYHA IV patients.**

|  | **ALL**  (n=375) | | **SURVIVOR**  (n=313) | | **NON-SURVIVOR**  (n=62) | | **P-VALUE**  Survivor vs. Non-survivor | |
| --- | --- | --- | --- | --- | --- | --- | --- | --- |
| **LEFT ATRIUM AND VENTRICLE** | | | | | | | | |
| EF (%) | 51±12 | | 51±12 | | 47±13 | | 0.07 | |
| LVGLS (%) | -14.6±5.0 | | -14.9±5.0 | | -13.4±4.9 | | 0.03 | |
| **AORTIC VALVE** | | | | | | | | |
| AV Vmax (m/s) | 4.1±0.8 | | 4.1±0.7 | | 4.0±0.9 | | 0.22 | |
| AV Area (cm^2^) | 0.7±0.2 | | 0.7±0.2 | | 0.7±0.2 | | 0.55 | |
| **RIGHT VENTRICLE** | | | | | | | | |
| TR gradient > 30 mmHg | 40.3% (147/362) | | 38.8% (118/304) | | 50.0% (29/58) | | 0.11 | |
| **CLINICAL RISK SCORE** | | | | | | | | |
| Eurolog II (%) | | 2.9 (IQR 2.8) | | 2.8 (IQR 2.5) | | 3.8 (IQR 4.4) | | 0.003 |

EF: Ejection Fraction, LVGLS: Left Ventricular Global Longitudinal Strain, AV: Aortic Valve, Vmax: Maximal Velocity, TR: Tricuspid Regurgitation.

Presented as mean±SD or fraction (%).

**Additional file 2: Table S2. Clinical characteristics of patients with preserved and impaired LVGLS**

|  | **LVGLS** ≤ -**14%**  (n=211) | **LVGLS** > -**14%**  (n=200) | **P-VALUE** |
| --- | --- | --- | --- |
| Age (years) | 80.2±6.8 | 80.1±7.3 | 0.91 |
| Female Sex | 53.6% (113/211) | 38.0% (76/200) | 0.002 |
| BMI (kg/m^2^) | 26.3±4.3 | 26.7±4.7 | 0.36 |
| Body Surface Area (m^2^) | 1.83±0.18 | 1.87±0.22 | 0.09 |
| Systolic Blood Pressure (mmHg) | 145±21 | 138±24 | 0.02 |
| Diastolic Blood Pressure (mmHg) | 75±11 | 74±13 | 0.26 |
| Creatinine Clearance (ml/min) | 59 (IQR 29) | 58 (QR 27) | 0.64 |
| Eurolog II (%) | 2.6 (IQR 2.2) | 3.9 (IQR 4.4) | 0.0001 |
| Hemodialysis | 1.0% (2/211) | 1.0% (2/200) | 0.96 |
| Previous Myocardial Infarction | 7.0% (14/199) | 17.2% (32/186) | 0.002 |
| Hypertension | 71.9% (138/192) | 73.1% (133/182) | 0.80 |
| COPD | 9.5% (20/211) | 22.5% (45/200) | 0.0003 |
| PAD | 12.4% (26/210) | 16.5% (33/200) | 0.24 |
| DM2 | 15.7% (30/191) | 20.1% (38/181) | 0.19 |
| NYHA I-II | 23.8% (50/210) | 21.1% (42/199) | 0.01 |
| NYHA III | 71.4% (150/210) | 65.8% (131/199) |  |
| NYHA IV | 4.8% (10/210) | 13.1% (26/199) |  |
| **MEDICATIONS** | | | |
| Statins | 55.9% (109/195) | 62.4% (113/181) | 0.20 |
| Beta Blockers | 41.6% (87/209) | 59.1% (117/198) | 0.0004 |
| Calcium Antagonists | 31.4% (66/210) | 18.2% (36/198) | 0.002 |
| ACE Inhibitors/ARB | 27.6% (58/210) | 39.4% (78/198) | 0.001 |
| Anticoagulant Treatment | 20.5% (43/210) | 38.4% (76/198) | 0.007 |
| Thrombocyte Inhibitors | 42.9% (90/210) | 32.3% (64/198) | 0.36 |
